# Multimorbidity trends in Catalonia, 2010–21: a population-based cohort study

**DOI:** 10.1093/ije/dyaf218

**Published:** 2026-01-02

**Authors:** Iñaki Permanyer, Jordi Gumà, Sergi Trias-Llimós, Aïda Solé-Auró

**Affiliations:** Centre for Demographic Studies (CED-CERCA), Universitat Autònoma de Barcelona, Bellaterra/Barcelona, Spain; ICREA, Passeig Lluís Companys 23, Barcelona, Spain; Centre for Demographic Studies (CED-CERCA), Universitat Autònoma de Barcelona, Bellaterra/Barcelona, Spain; Centre for Demographic Studies (CED-CERCA), Universitat Autònoma de Barcelona, Bellaterra/Barcelona, Spain; Department of Political and Social Sciences, DemoSoc Research Group, Universitat Pompeu Fabra, Barcelona, Spain; Department of Political and Social Sciences, DemoSoc Research Group, Universitat Pompeu Fabra, Barcelona, Spain

**Keywords:** aging, multimorbidity, inequalities, mortality, health registers

## Abstract

**Background:**

With rising longevity, multimorbidity is an increasingly important challenge for healthcare systems. We describe trends in the prevalence and incidence of multimorbidity across socioeconomic groups in Catalonia.

**Methods:**

We use a random sample of 1 551 126 individuals (22% of the Catalan population, for whom we have the complete primary care health records) and follow them from 2010 until 2021. We document the age- and sex-specific prevalence and incidence of multimorbidity stratifying by income groups and birth cohorts. Logistic regression models are used to estimate the association between multimorbidity and mortality.

**Results:**

Between 2010 and 2021, the prevalence of multimorbidity, higher among women, has increased for both sexes and all cohorts in our analysis. Importantly, each cohort attains the same ages, with higher multimorbidity prevalence than their predecessors had 10 years ago. Older generations are mostly affected by degenerative diseases, while younger age groups are more affected by mental health problems. Incidence tends to be higher among the older cohorts across all adult ages. We observe a strong socioeconomic gradient, with lower-income individuals experiencing worse multimorbidity prevalence and incidence. Such a gradient is persistent and becomes more pronounced at the end of the study period. Across all age groups, individuals experiencing multimorbidity have a higher risk of dying than those who do not.

**Conclusion:**

The documented increases in multimorbidity alongside its socioeconomic gradients suggest that preventive strategies are urgently needed to defer or prevent its onset and slow its progression—especially among younger generations.

Key Messages

- This paper describes the multimorbidity prevalence and incidence trends across socioeconomic groups between 2010 and 2021 by using the “Health Inequalities” cohort dataset—a representative sample covering 22% of the Catalan population in 2005 that is linked to mortality registers.
- We found evidence of worsening health across the cohorts, with a higher prevalence of multimorbidity in younger cohorts at equivalent ages and a strong socioeconomic gradient.
- Our findings highlight significant challenges for future healthcare systems, but they also provide evidence that investing in improving individuals’ living conditions and other social determinants of health, through efficient and equitable policies, can have an impact in preventing or delaying the onset of multimorbidity and slowing its progression to more severe stages and death.

## Introduction

Multimorbidity (i.e. the coexistence of two or more chronic conditions for the same individual) is one of the major challenges in the provision and structure of healthcare systems, particularly in low-mortality countries [1]. In a context of rising life expectancy, it is increasingly likely that individuals will develop different chronic conditions as they survive to higher ages—an issue that can impose an individual and collective burden given its association with a set of factors, including higher healthcare utilization, mortality risks [2–4], or early retirement [5]. In this regard, it is extremely important to understand the disease-specific composition of multimorbidity and how it varies over time across cohorts and socioeconomic groups [6].

Multimorbidity assessments are sensitive to the list of diseases and the source of data used in the analyses (e.g. health register data, medical prescriptions, health surveys) [7, 8]. Irrespective of the followed approach, previous studies suggest that multimorbidity has increased over the last decades in low-mortality settings, including Scotland, Sweden, the USA, and the UK [1, 9–11]. Some of these studies often identify a clear socioeconomic gradient, whereby lower-socioeconomic-status (SES) groups tend to experience higher multimorbidity when using both area-level SES indicators [1, 12, 13] and individual-level indicators [13–15]. Additionally, empirical evidence links multimorbidity to higher mortality risks, but such an association varies considerably by space, time, cohort, and multimorbidity type [1, 4].

In Spain, one of the most longevous countries in the world, the evidence on patterns of multimorbidity by SES based on exhaustive register data is scarce and mostly limited to a few region-specific studies assessing area-level inequalities [16, 17]. Some other studies have used socioeconomic variables as a control, without being the main focus of the analysis [18, 19]. In addition, most of these studies adopted a cross-sectional approach, limiting our understanding of multimorbidity over time. A recent longitudinal study investigated (multi-)morbidity trajectories in a region within Spain, but only examined the trajectories of those who died [20].

Using medical records from Catalonia’s public health system (a Spanish region with ∼8 million inhabitants and with high life expectancy), we adopted a birth cohort perspective with three main objectives. First, we explore the multimorbidity prevalence and incidence trends between 2010 and 2021. Second, we describe the multimorbidity prevalence and incidence socioeconomic gradients. Finally, we assess the mortality risks associated with multimorbidity.

## Methods

### Data sources

We used the “Health Inequalities” (HEALIN) cohort [21]—a population-based dataset drawing on primary care and hospital record diagnostics for a random sample of 1 551 126 individuals (accounting for 22% of the Catalan population) who were followed up from 2005 to 2021. This sample was representative in terms of age, sex, and geographical distribution. All data within the HEALIN cohort are anonymized and comply with Spanish regulations regarding observational studies. We used primary care diagnostics and all-cause mortality information. The list of 401 diseases considered in the database is shown in Supplementary Table 1. Follow-up terminations may have occurred due to death or change of residence after 2005.

To minimize biases that might have arisen from lack of information before 2005, a 5-year washout period (2005–9) was used, setting the observation window from 2010 to 2021. Employing a washout period has minimized the overestimation of prevalent cases in truncated registers [22]. Only those individuals without diagnoses during the washout were considered to have been disease-free in 2010. The results exclude those born before 1930 and between 2000 and 2004 due to small cohort sizes resulting in volatile results.

### Population characteristics

Among the >1.5 million individuals included in the 2005 sample, 50.3% were women and 65.7% resided in the region including the capital city of Barcelona. In the same year, the mean age was 42.3 years for women and 39.1 years for men.

In addition to demographic characteristics, we used data on pharmacy copayment to derive individuals’ income levels (measured in three brackets: <€18 000 per year, between €18 000 and €100 000 per year, and >€100 000 per year) for those who were part of the active population (retired individuals, long-term unemployed, or those disabled due to health reasons were not included in this classification). This information was only available from 2014 onwards. In 2014, 66.2% of individuals belonged to the lower-income group, 32.8% to the middle-income group, and 1.0% to the higher-income group.

### Definition of multimorbidity

We used two alternative definitions of multimorbidity, which allowed a more nuanced description of populations’ health. “Basic multimorbidity” was defined as the co-occurrence of two or more chronic diseases. “Complex multimorbidity” was defined as the occurrence of three or more chronic diseases from the same list affecting at least three different body systems simultaneously. In both cases, individuals were considered to have the disease whenever they received a diagnosis. Furthermore, we assessed the sensitivity of our estimates by using the diseases found simultaneously in the HEALIN cohort and in a recent previous study [1].

### Statistical analysis

Standard descriptive statistics are used to describe the multimorbidity trends, which are disaggregated by birth cohort (e.g. 1930–39, …, 1990–99), sex, age, and income group. Prevalence is defined as the ratio between the number of individuals complying with the corresponding definition of multimorbidity for a specific year and the number of persons alive in that year. Supplementary Fig. 1 illustrates how the multimorbidity prevalence was calculated across ages for different cohorts. The incidence was calculated based on the individuals who transitioned to “multimorbidity” (basic or complex) in a specific year. The denominator encompassed all individuals alive in the same year who had not experienced the transition to multimorbidity in previous years. The age at multimorbidity onset was determined by the first year in which individuals met the corresponding definition of multimorbidity. Finally, we ran logistic regression models with all-cause mortality as the dependent variable and multimorbidity prevalence adjusted for year, region, and cohort as independent variables, separately by gender.

## Results

**Figure 1.**
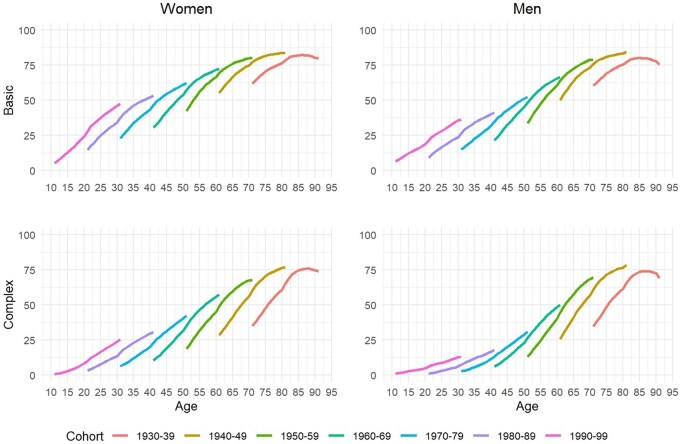
Trends in multimorbidity prevalence by age groups/cohorts (women and men separately) between 2010 and 2021. *Source*: Authors’ elaboration based on the HEALIN cohort database.

Fig. 1 shows the trends in basic and complex multimorbidity prevalence by age and sex between 2010 and 2021 for seven cohorts born between 1930 and 1999 (given the large sample size, the confidence intervals are very narrow, so they are not shown here). The prevalence of basic and complex multimorbidity increased steadily during the study period for women and men—except for the older cohort, which exhibited an inverted U trend. Older individuals experienced higher levels of basic and complex multimorbidity, while, among younger cohorts, the prevalence was lower but increased at a fast pace over time. Overall, the disease-accumulation trajectories among the different cohorts shown in Fig. 1 were S-shaped, with fast increases at young and adult ages, slowing down at older ages.

The intensity of health decline with age appeared to vary by birth cohort. Interestingly, at a given age, the multimorbidity prevalence was higher among younger cohorts for basic and complex multimorbidity, both for women and for men. For instance, the prevalence of basic multimorbidity among women born between 1980 and 1989 was 37% when they reached the age of 31 years, but this prevalence increased to 47% among women who were born one decade later (i.e. between 1990 and 1999) when they reached the same age. Our findings indicate that the median age at basic multimorbidity onset decreased between 2010 and 2021, from 47 to 45 years among women and from 48 to 45 among men (see Supplementary Fig. 2), and similar findings were observed for complex multimorbidity.

Table 1 shows the prevalence in 2021 of large groups of diseases (classified by International Statistical Classification of Diseases and Related Health Problems, 10th Revision chapters) for the cohorts shown in Fig. 1. Older generations (e.g. those individuals born between 1930 and 1939) had a relatively higher prevalence for most diseases, including endocrine, nutritional, and metabolic diseases, and circulatory and musculoskeletal systems (i.e. with prevalence levels typically >50%). Younger cohorts were strongly affected by mental health disorders (with prevalence levels of 27% and 21% for women and men, respectively), which was the most-diagnosed group of diseases among this cohort after diseases of the musculoskeletal system (39% and 34%, respectively).

**Table 1.** Prevalence of diseases grouped by International Statistical Classification of Diseases and Related Health Problems, 10th Revision chapters by sex for different cohorts in 2021.

Women							
	1930–39	1940–49	1950–59	1960–69	1970–79	1980–89	1990–99
**Chapter I**	2.79	2.26	1.50	1.02	0.49	0.27	0.08
**Chapter II**	17.04	14.60	10.68	6.57	2.91	1.05	0.43
**Chapter III**	24.59	14.29	10.57	15.03	14.20	10.63	9.03
**Chapter IV**	60.12	64.15	57.51	40.55	24.51	18.22	15.51
**Chapter V**	45.78	42.25	44.17	43.73	39.21	36.40	27.40
**Chapter VI**	20.88	19.22	18.54	17.83	13.60	10.93	8.51
**Chapter VII**	25.64	22.19	13.87	8.18	4.15	2.45	4.02
**Chapter VIII**	29.00	24.08	18.08	13.17	9.12	6.57	4.40
**Chapter IX**	73.59	66.48	50.16	34.10	23.34	19.72	19.35
**Chapter X**	11.22	10.21	8.26	5.99	4.86	4.44	5.16
**Chapter XI**	17.50	18.53	14.88	8.72	3.58	1.90	1.04
**Chapter XII**	3.94	1.21	0.56	0.37	0.20	0.11	0.07
**Chapter XIII**	75.13	75.72	70.57	62.40	56.77	50.59	39.31
**Chapter XIV**	29.70	14.78	6.16	3.29	1.76	0.83	0.31

Men							
	1930–39	1940–49	1950–59	1960–69	1970–79	1980–89	1990–99
**Chapter I**	2.18	2.00	1.67	1.77	0.91	0.44	0.12
**Chapter II**	27.46	21.85	11.24	3.99	1.37	0.72	0.42
**Chapter III**	23.82	14.73	7.09	3.56	2.01	1.49	2.04
**Chapter IV**	53.15	58.77	51.63	36.77	21.47	11.23	10.91
**Chapter V**	33.68	33.22	34.58	33.45	29.98	28.69	20.72
**Chapter VI**	17.74	14.69	11.01	8.09	5.98	4.76	4.51
**Chapter VII**	24.41	20.48	12.15	6.67	3.10	1.63	2.93
**Chapter VIII**	24.51	20.99	14.82	9.10	5.95	4.10	2.81
**Chapter IX**	71.94	67.67	52.34	33.25	18.73	14.35	15.08
**Chapter X**	18.34	15.78	10.22	5.44	3.82	3.58	5.56
**Chapter XI**	16.45	18.43	15.51	9.66	4.71	2.39	1.03
**Chapter XII**	2.93	1.28	0.71	0.40	0.22	0.10	0.06
**Chapter XIII**	66.10	66.71	59.97	52.12	45.14	39.89	34.23
**Chapter XIV**	55.54	52.58	33.44	13.53	3.75	1.33	0.58

Chapter I: Certain infectious and parasitic diseases; Chapter II: Neoplasms; Chapter III: Diseases of the blood and blood-forming organs and certain disorders involving the immune mechanism; Chapter IV: Endocrine, nutritional and metabolic diseases; Chapter V: Mental and behavioural disorders; Chapter VI: Diseases of the nervous system; Chapter VII: Diseases of the eye and adnexa; Chapter VIII: Diseases of the ear and mastoid process; Chapter IX: Diseases of the circulatory system; Chapter X: Diseases of the respiratory system; Chapter XI: Diseases of the digestive system; Chapter XII: Diseases of the skin and subcutaneous tissue; Chapter XIII: Diseases of the musculoskeletal system and connective tissue; Chapter XIV: Diseases of the genitourinary system.

*Source:* Authors’ elaboration based on the HEALIN cohort database.

**Figure 2.**
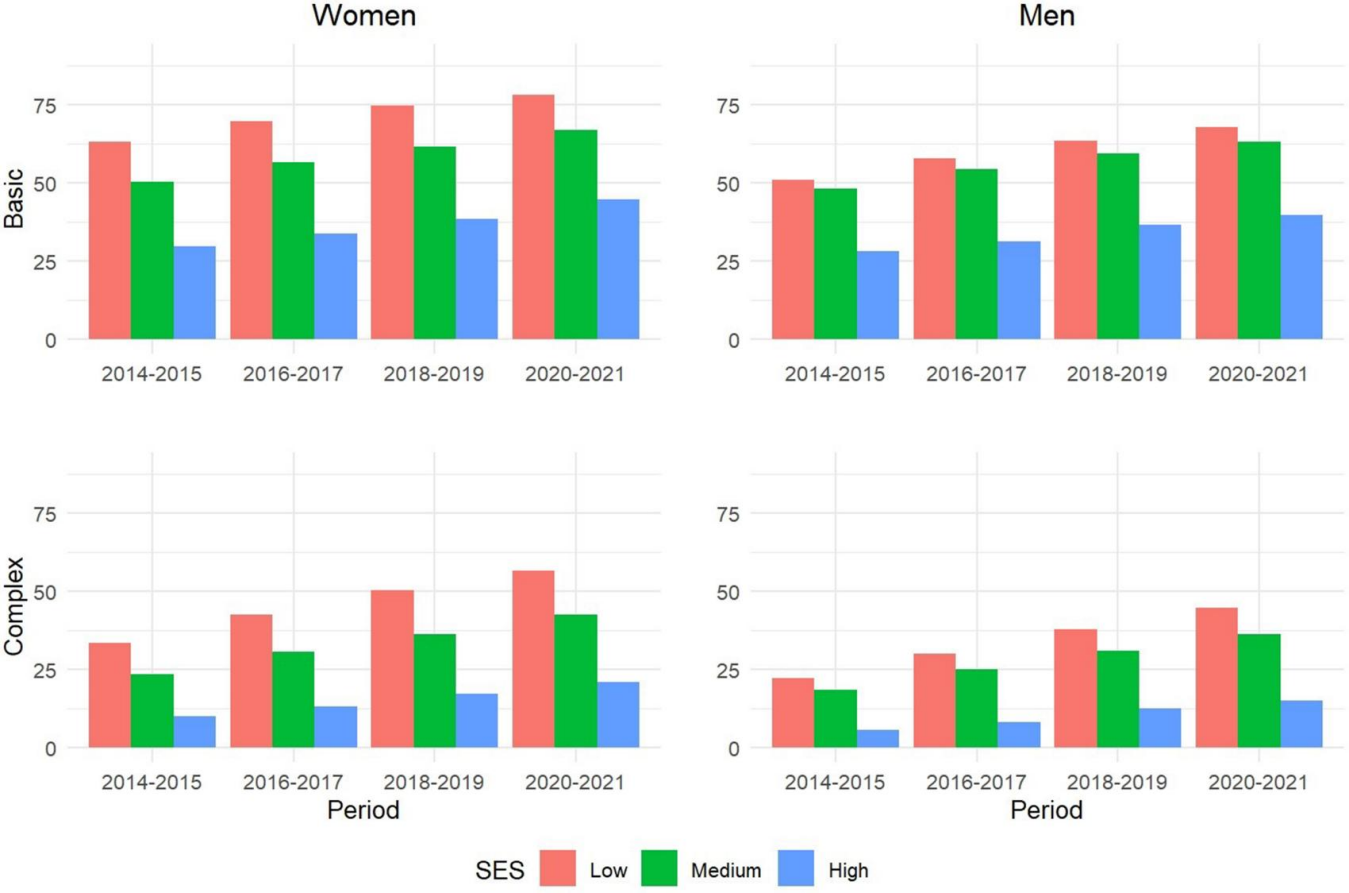
Prevalence of basic and complex multimorbidity across SES groups by age/cohort and sex between 2014 and 2021. *Source*: Authors’ elaboration based on the HEALIN cohort database.

Fig. 2 documents the levels and trends in basic and complex multimorbidity for women and men born between 1960 and 1979 by income levels, which were always higher among women than among men across all years and income groups. The lowest-income group exhibited a higher prevalence of basic and complex multimorbidity than the middle- and, especially, the higher-income groups. The basic multimorbidity for low-income individuals went from 60% to 78% between 2014 and 2021 among women and from 48% to 70% among men. Similar trends, but with lower levels, were observed among high-income individuals (increasing from 26% to 41% among women and from 17% to 32% among men). As regards complex multimorbidity, its prevalence increased across all income groups, but at different rates. While it increased vigorously from 34% in 2014 to 57% in 2021 among low-income women (and from 22% to 45% among men), it only increased from 10% to 21% among their high-income counterparts (and from 6% to 15% among men).

**Figure 3.**
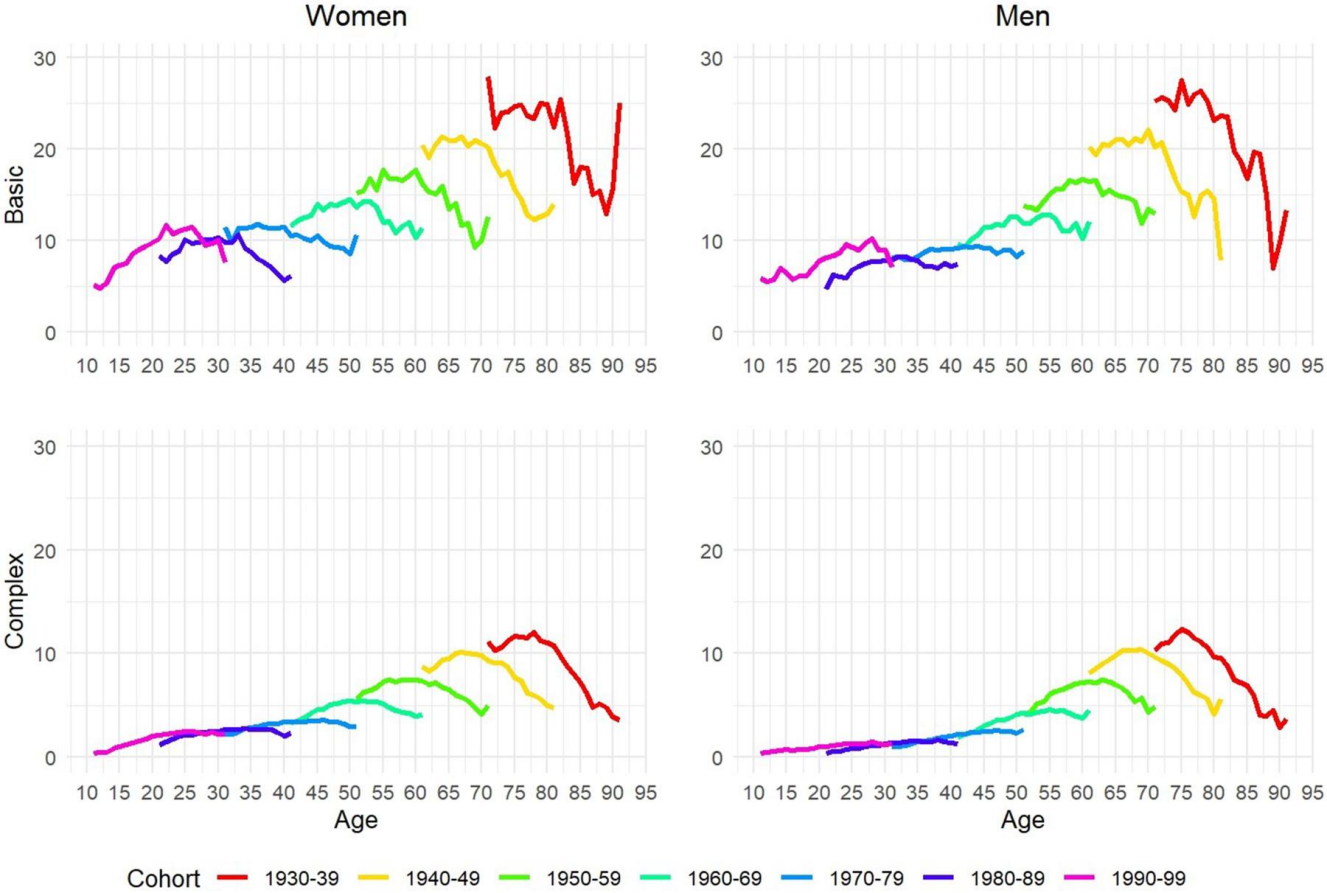
Multimorbidity incidence by cohort and sex, according to basic and complex multimorbidity. *Source*: Authors’ elaboration based on the HEALIN cohort database.

There is a clear gradient in the incidence of basic and complex multimorbidity across cohorts by age and sex (Fig. 3), whereby older cohorts tend to experience higher multimorbidity incidence than younger cohorts. Among those born between 1990 and 1999, the incidence of basic multimorbidity hovered at ∼10 per 100 individuals, while, for those born between 1930 and 1939, it was at least twice as high. For complex multimorbidity, the incidence moved from ∼2 per 100 among the youngest cohort to ∼8 per 100 among the oldest cohort. Overall, the incidence of multimorbidity tends to be higher among women compared with among men. The shape of the different multimorbidity incidence curves by age varied across cohorts: it looked to be increasing over age for younger cohorts, was inversely U-shaped for many cohorts, and was declining for the older cohorts (see Fig. 3).

The main patterns shown in Figs 1 and 3 regarding the prevalence and incidence of multimorbidity across birth cohorts are robust to alternative specifications of basic and complex multimorbidity when employing the conditions in our dataset that are also included in recent studies [1] (see Supplementary Figs S3 and S4).

**Figure 4.**
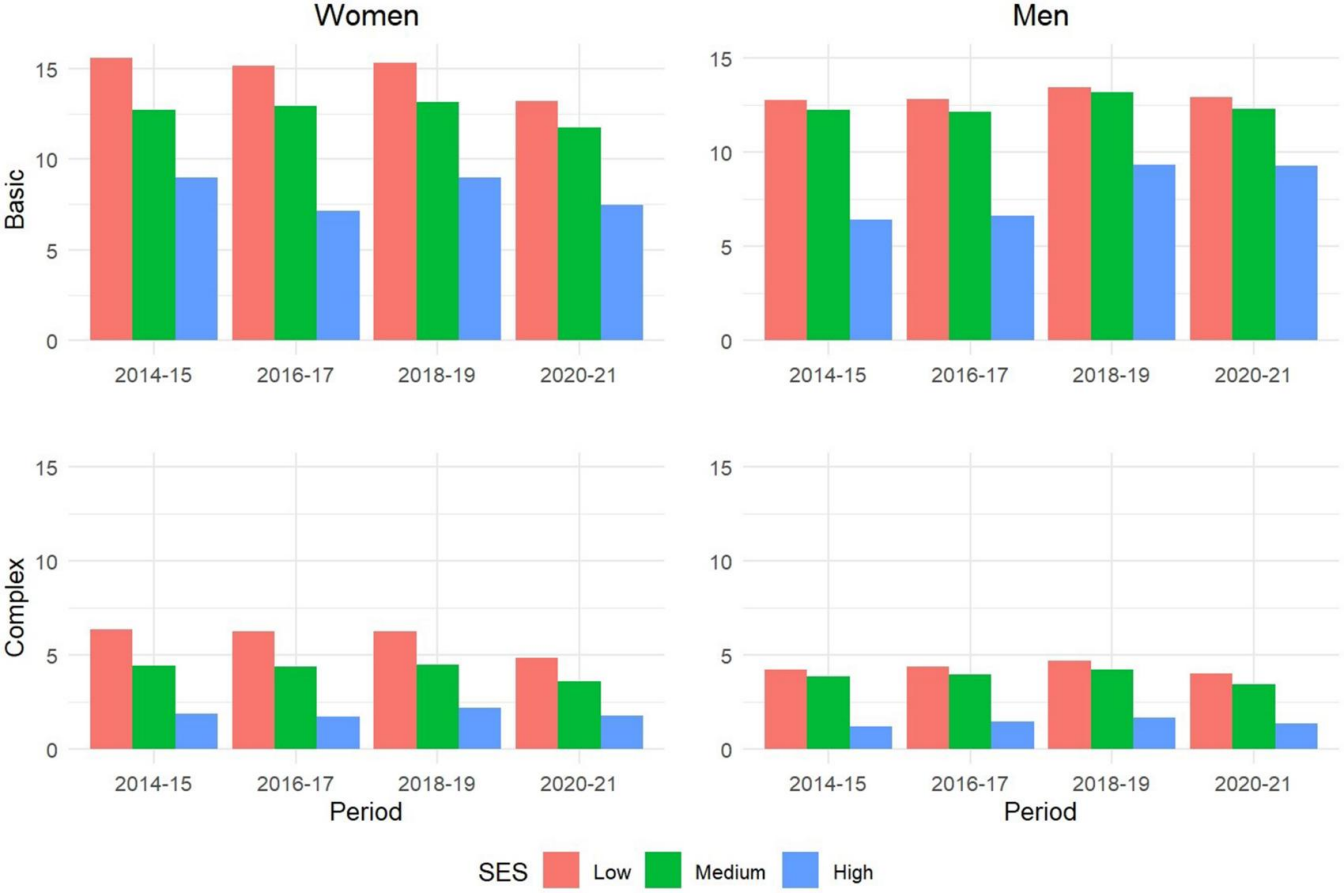
Multimorbidity incidence by sex and SES levels according to basic and complex multimorbidity. *Source*: Authors’ elaboration based on the HEALIN cohort database.

The SES gradient is observed again in the multimorbidity incidence between 2014 and 2021 (Fig. 4). Lower-income groups experienced higher multimorbidity incidence than their higher-income counterparts for all years between 2014 and 2021—for both basic and complex multimorbidity, and for women and men alike.

**Figure 5.**
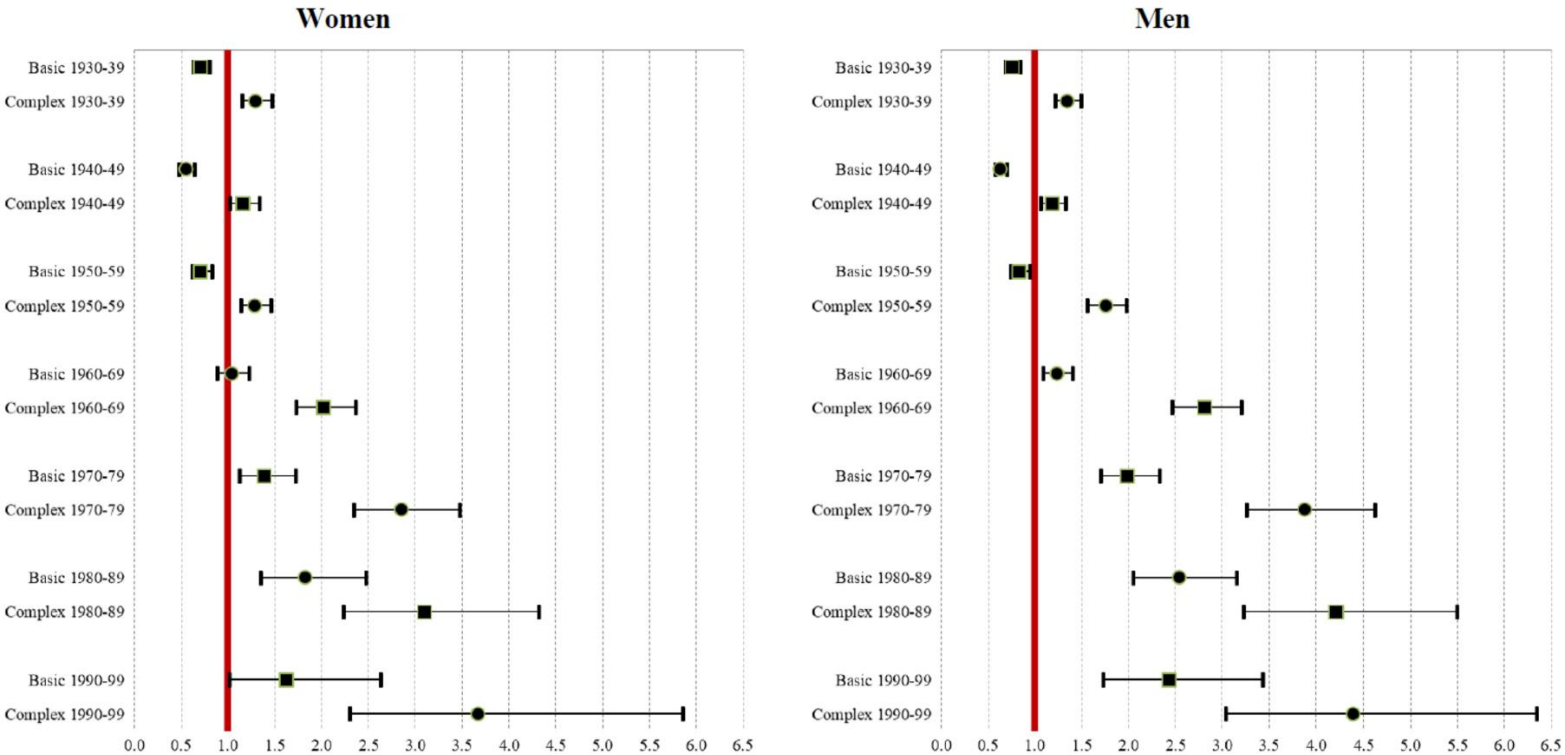
Relative risk of dying for individuals experiencing basic and complex multimorbidity for women (left panel) and men (right panel). *Source*: Authors’ elaboration based on the HEALIN cohort database.

Conducting logistic regressions for each cohort of birth in our study (adjusted for year, cohort, and region) separately for women and men, we found that those individuals experiencing complex multimorbidity had higher risks of dying than those individuals not experiencing multimorbidity (Fig. 5 and Supplementary Table S2). For instance, for the 1990–99 birth cohort, the relative risk for women was 3.67 [95% confidence interval (CI) 2.30, 5.86] and 4.39 (95% CI 3.03, 6.35) for men, while, for the 1930–39 birth cohort, such relative risks were 1.30 (95% CI 1.15, 1.46) and 1.35 (95% CI 1.22, 1.49), respectively. In addition, individuals experiencing complex multimorbidity had higher risks of dying than those who only experienced basic multimorbidity. Among older cohorts, experiencing basic multimorbidity was associated with lower mortality risks.

## Discussion

In this study, we have documented the patterns of multimorbidity in Catalonia between 2010 and 2021. We observed notable increases in the prevalence of multimorbidity across ‘all’ generations, not only the older age groups. In addition, the prevalence of multimorbidity was higher among women. As expected, the health profiles of multimorbid individuals differed considerably across cohorts. While older multimorbid individuals were often affected by degenerative diseases or diseases affecting a more variegated set of body systems (such as hypertension, urinary incontinence, or osteoarthritis), the younger people were more strongly affected by mental health disorders. The heterogeneity in multimorbidity profiles is important because, under its label, there are distinct epidemiological characteristics with specific risk factors, each requiring tailored prevention and management policies.

Inspecting the patterns of disease accumulation by age through the levels of multimorbidity prevalence across cohorts, we observed that they followed an S-shaped trajectory (see Fig. 1). Importantly, (i) each cohort reached a certain age with higher cumulative multimorbidity than did the preceding generation some years before (a trend aligned with recent studies in other countries [9–11, 22–26] and this has been referred to as a “generational health drift” [27] and (ii) young cohorts were already in a quickly expanding phase of multimorbidity, with fast increases in short time periods. These findings point towards an important health policy goal to flatten the disease-accumulation curves—especially among younger generations, for which there is more room for improvement.

The finding that nearly 50% of women belonging to the younger generation (i.e. those born between 1990 and 1999) complied with the definition of basic multimorbidity in 2021 is a matter of concern. Beyond dorsalgia (the most prevalent condition across all generations), the main cause of this high prevalence was mental health, which is an increasingly prominent public health concern that has been on the rise during the last decades. Importantly, Spain is among the world countries with the highest prescription levels of benzodiazepine and other sedatives [28]—a trend that has greatly increased since the outbreak of COVID-19 [29] and which, unfortunately, affects many other age groups as well (i.e. not only the youngest generation). Policies aiming at addressing this important issue should receive the highest priority.

Key to our findings are the large and widening socioeconomic differentials in multimorbidity measures that have been identified both for women and for men. These findings are consistent with those reported in previous studies documenting diverging trends in health outcomes across SES groups (with higher-SES individuals systematically outperforming lower-SES people [1, 30, 31]). The results we observed across generations, SES groups, and over time suggest that increasing multimorbidity trends are likely to continue in the coming years. In turn, differences across SES groups suggest that multimorbidity is not solely a matter of age, indicating that public health policies targeting social determinants of health could help delay or prevent the onset of certain diseases [32]. Likewise, delaying or preventing the transition from basic to complex multimorbidity should be an important health policy goal to reduce the burden associated with individuals’ severe health deterioration.

An important strength of our study was the possibility of documenting multimorbidity patterns directly by using the official medical records for the whole population of Catalonia through a huge representative sample of >1.5 million (22% of the entire population). In this way, the estimates were very reliable and allowed the possibility of conducting highly detailed analyses. Another strength of our study was the possibility of linking multimorbidity with mortality information at the population level, which is quite uncommon outside Scandinavian countries [33]. Our findings underscore the distinct association with mortality risk depending on the definition of multimorbidity—thus enabling a more nuanced characterization of population health profiles.

It is difficult to ascertain whether the increases in multimorbidity documented in this study were attributable to “true changes” in the underlying distribution of health, to a growing tendency towards overdiagnosis among health professionals [34], or to changes in individuals’ health-seeking behavior. In turn, such behavior might have been influenced by public health prevention programs, such as screening campaigns promoting the purported benefits of early diagnoses. Additionally, technological improvements (e.g. artificial intelligence-aided diagnostic software) could have facilitated very early diagnoses that might have helped in delaying the progression of diseases to more severe stages. Unfortunately, with the data used in our analyses, it was not possible to single out the influence of these factors separately.

Our findings on increasing multimorbidity prevalence over time indicate an increasing demand on public health care, especially among younger generations—a result that aligns with those of previous longitudinal cohort and other studies [1, 9–11, 23]. Investigating the risk factors explaining such disease-accumulation trajectories should be the focus of future work to find effective prevention strategies.

This study comes with several limitations. First, as we tracked a representative sample of the Catalan population from 2005 onwards, we lacked information on the health profiles of these individuals prior to the beginning of our observation period. For this reason, we implemented a 5-year washout period (2005–9), which should have improved the accuracy of our prevalence and incidence estimates.

Second, the proxy we used to estimate individuals’ SES status had a broad middle-income interval, and the top-income interval only included a small minority of highly selected individuals. Additionally, when inspecting SES health differentials, we had to restrict our attention to the age range between 30 and 60 years so as to include individuals who had completed their education and had not reached the average retirement age in Spain (62 years in 2022). Despite these limitations, we have been able to identify a clear SES gradient in multimorbidity that should be taken into consideration in the elaboration of prospective health policies.

Lastly, our dataset only included information from the public health sector, and not from the private sector. While this could have potentially biased our level estimates, it is very unlikely that it affected the reported time trends. In addition, the majority of diagnoses ended up being reported in the public sector as well, owing to pharmacy prescriptions and social security benefits.

Interestingly, we did not observe any remarkable changes in the prevalence of multimorbidity for the COVID-19 outbreak years (2020 and 2021). We only observed a small dip in the multimorbidity incidence levels in 2020 across all generations, which was most likely driven by the inaccessibility to hospitals and health centers generated by the lockdown and other preventive measures to restrict individuals’ mobility. In 2021, the multimorbidity incidence levels went back to those that were observed in 2019 across all age groups.

To conclude, our findings indicate that basic and complex multimorbidity have been on the rise in Catalonia since 2010, whilst the age at onset has quickly declined. Importantly, each generation seemed to attain different ages with higher multimorbidity prevalence than did the previous generation some years before, and women tended to fare worse than men. In addition, we found a clear socioeconomic gradient, with lower-income individuals performing systematically worse than their higher-income peers. This poses considerable challenges for prospective healthcare systems, but also provides evidence that investing in improving individuals’ living conditions and other social determinants of health through efficient and equitable policies (e.g. affordable housing and/or better working conditions) can have a strong impact on delaying or preventing the onset of multimorbidity and its progression towards more severe stages and death.

## Ethics approval

This study was performed in line with the principles of the Declaration of Helsinki. Approval was granted by the Ethics Committee of the Universitat Autònoma de Barcelona (02/21/2025/20250221CS) for the HEALIN data.

## Supplementary Material

dyaf218_Supplementary_Data

## Data Availability

For confidentiality reasons, the HEALIN cohort data are not publicly accessible. Anyone interested in accessing the data is encouraged to contact Iñaki Permanyer (ipermanyer@ced.uab.es) or Aïda Solé-Auró (aida.sole@upf.edu).

## References

[1] Head A , Fleming K, Kypridemos C, Schofield P, Pearson-Stuttard J, O’Flaherty M. Inequalities in incident and prevalent multimorbidity in England, 2004–19: a population-based, descriptive study. Lancet Healthy Longev 2021;2:e489–97–e497. 36097998 10.1016/S2666-7568(21)00146-X

[2] Glynn LG , Valderas JM, Healy P et al The prevalence of multimorbidity in primary care and its effect on health care utilization and cost. Fam Pract 2011;28:516–23. 21436204 10.1093/fampra/cmr013

[3] Willadsen T , Siersma V, Nicolaisdóttir D et al Multimorbidity and mortality: a 15-year longitudinal registry-based nationwide Danish population study. J Comorb 2018;8:2235042X18804063. X1880406. 10.1177/2235042X18804063 PMC6194940 30364387

[4] Nunes BP , Flores TR, Mielke GI, Thumé E, Facchini LA. Multimorbidity and mortality in older adults: a systematic review and meta-analysis. Arch Gerontol Geriatr 2016;67:130–8. 27500661 10.1016/j.archger.2016.07.008

[5] Van Zon SKR , Reijneveld SA, Galaurchi A, Mendes De Leon CF, Almansa J, Bültmann U. Multimorbidity and the transition out of full-time paid employment: a longitudinal analysis of the health and retirement study. J Gerontol B Psychol Sci Soc Sci 2020;75:705–15. 31083712 10.1093/geronb/gbz061 PMC7768699

[6] Violan C , Foguet-Boreu Q, Flores-Mateo G et al Prevalence, determinants and patterns of multimorbidity in primary care: a systematic review of observational studies. PLoS One 2014;9:e102149. 25048354 10.1371/journal.pone.0102149 PMC4105594

[7] Ho IS-S , Azcoaga-Lorenzo A, Akbari A et al Examining variation in the measurement of multimorbidity in research: a systematic review of 566 studies. Lancet Public Health 2021;6:e587–97–e597. 34166630 10.1016/S2468-2667(21)00107-9

[8] Violán C , Foguet-Boreu Q, Hermosilla-Pérez E et al Comparison of the information provided by electronic health records data and a population health survey to estimate prevalence of selected health conditions and multimorbidity. BMC Public Health 2013;13:251–10. 23517342 10.1186/1471-2458-13-251 PMC3659017

[9] Bishop NJ , Haas SA, Quiñones AR. Cohort trends in the burden of multiple chronic conditions among aging U.S. adults. J Gerontol B Psychol Sci Soc Sci 2022;77:1867–79. 35642746 10.1093/geronb/gbac070 PMC9535783

[10] Canizares M , Hogg-Johnson S, Gignac MAM, Glazier RH, Badley EM. Increasing trajectories of multimorbidity over time: birth cohort differences and the role of changes in obesity and income. J Gerontol B Psychol Sci Soc Sci 2018;73:1303–14. 28199711 10.1093/geronb/gbx004

[11] Ribe E , Cezard GI, Marshall A, Keenan K. Younger but sicker? Cohort trends in disease accumulation among middle-aged and older adults in Scotland using health-linked data from the Scottish Longitudinal Study. Eur J Public Health 2024;34:ckae062–703. 10.1093/eurpub/ckae062 PMC11293808 38604658

[12] Barnett K , Mercer SW, Norbury M, Watt G, Wyke S, Guthrie B. Epidemiology of multimorbidity and implications for health care, research, and medical education: a cross-sectional study. Lancet 2012;380:37–43. 22579043 10.1016/S0140-6736(12)60240-2

[13] Knies G , Kumari M. Multimorbidity is associated with the income, education, employment and health domains of area-level deprivation in adult residents in the UK. Sci Rep 2022;12:7280. 35508678 10.1038/s41598-022-11310-9 PMC9068903

[14] Schiøtz ML , Stockmarr A, Høst D, Glümer C, Frølich A. Social disparities in the prevalence of multimorbidity–a register-based population study. BMC Public Health 2017;17:422. 28486983 10.1186/s12889-017-4314-8 PMC5424300

[15] Mondor L , Cohen D, Khan AI, Wodchis WP. Income inequalities in multimorbidity prevalence in Ontario, Canada: a decomposition analysis of linked survey and health administrative data. Int J Equity Health 2018;17:90. 29941034 10.1186/s12939-018-0800-6 PMC6019796

[16] Alvarez-Galvez J , Ortega-Martin E, Ramos-Fiol B, Suarez-Lledo V, Carretero-Bravo J. Epidemiology, mortality, and health service use of local-level multimorbidity patterns in South Spain. Nat Commun 2023;14:7689. 38001107 10.1038/s41467-023-43569-5 PMC10673852

[17] Violán C , Foguet-Boreu Q, Roso-Llorach A et al Burden of multimorbidity, socioeconomic status and use of health services across stages of life in urban areas: a cross-sectional study. BMC Public Health 2014;14:530. 24885174 10.1186/1471-2458-14-530 PMC4060853

[18] Monterde D , Vela E, Clèries M, Garcia-Eroles L, Roca J, Pérez-Sust P. Multimorbidity as a predictor of health service utilization in primary care: a registry-based study of the Catalan population. BMC Fam Pract 2020;21:39. 32066377 10.1186/s12875-020-01104-1 PMC7026948

[19] Violán C , Fernández-Bertolín S, Guisado-Clavero M et al Five-year trajectories of multimorbidity patterns in an elderly Mediterranean population using Hidden Markov Models. Sci Rep 2020;10:16879. 33037233 10.1038/s41598-020-73231-9 PMC7547668

[20] Sol J , Ortega-Bravo M, Portero-Otín M et al Human lifespan and sex-specific patterns of resilience to disease: a retrospective population-wide cohort study. BMC Med 2024;22:17. 38185624 10.1186/s12916-023-03206-w PMC10773063

[21] Solé-Auró A , Gumà-Lao J, Trias-Llimós S, Carreño Serra A, Martínez-Cerdá J-F, Permanyer I. Cohort Profile: Health Inequalities in Catalonia (the HEALIN cohort). Int J Epidemiol 2025;54:dyaf129. 40694832 10.1093/ije/dyaf129

[22] Plana-Ripoll O , Momen NC, Gallego-Alabanda D et al Impact of washout duration to account for left truncation in register-based epidemiologic studies: Estimating the risk of mental disorders. Epidemiology (Fairfax) 2025;36:719–29. 10.1097/EDE.0000000000001905 PMC13166074 40815060

[23] Zheng H , Echave P. Are recent cohorts getting worse? Trends in US adult physiological status, mental health, and health behaviors across a century of birth cohorts. Am J Epidemiol 2021;190:2242–55. 33738469 10.1093/aje/kwab076 PMC8799895

[24] Freedman VA , Spillman BC, Andreski PM et al Trends in late-life activity limitations in the United States: an update from Five National Surveys. Demography 2013;50:661–71. 23104207 10.1007/s13524-012-0167-z PMC3586750

[25] Martin LG , Freedman VA, Schoeni RF, Andreski PM. Trends in disability and related chronic conditions among people ages fifty to sixty-four. Health Aff (Millwood) 2010;29:725–31. 20368601 10.1377/hlthaff.2008.0746 PMC2874878

[26] Seeman TE , Merkin SS, Crimmins EM, Karlamangla AS. Disability trends among older Americans: National Health and Nutrition Examination Surveys, 1988–1994 and 1999–2004. Am J Public Health 2010;100:100–7. 19910350 10.2105/AJPH.2008.157388 PMC2791257

[27] Gimeno L , Goisis A, Dowd JB, Ploubidis GB. Cohort differences in physical health and disability in the United States and Europe. J Gerontol B Psychol Sci Soc Sci 2024;79:gbae113. 38898719 10.1093/geronb/gbae113 PMC11272052

[28] Huerta C , Abbing‐Karahagopian V, Requena G et al Exposure to benzodiazepines (anxiolytics, hypnotics and related drugs) in seven European electronic healthcare databases: a cross‐national descriptive study from the PROTECT‐EU Project. Pharmacoepidemiol Drug Saf 2016;25 Suppl 1:56–65. 26149383 10.1002/pds.3825

[29] Racine N , McArthur BA, Cooke JE, Eirich R, Zhu J, Madigan S. Global prevalence of depressive and anxiety symptoms in children and adolescents during COVID-19: a meta-analysis. JAMA Pediatr 2021;175:1142–50. 34369987 10.1001/jamapediatrics.2021.2482 PMC8353576

[30] Tetzlaff J , Epping J, Sperlich S, Eberhard S, Stahmeyer JT, Geyer S. Widening inequalities in multimorbidity? Time trends among the working population between 2005 and 2015 based on German health insurance data. Int J Equity Health 2018;17:103. 30012163 10.1186/s12939-018-0815-z PMC6048702

[31] Chan MS , Van Den Hout A, Pujades-Rodriguez M et al Socio-economic inequalities in life expectancy of older adults with and without multimorbidity: a record linkage study of 1.1 million people in England. Int J Epidemiol 2019;48:1340–51. 30945728 10.1093/ije/dyz052 PMC6693817

[32] Ruiz Alvarez M , Aginagalde Llorente AH, Del Llano Señarís JE. Los determinantes sociales de la salud en España (2010-2021): una revisión exploratoria de la literatura. *Rev Esp Salud Pública* 2022;96:e1–e58. 35582978

[33] Smith Jervelund S , De Montgomery CJ. Nordic registry data: value, validity and future. Scand J Public Health 2020;48:1–4. 31985364 10.1177/1403494819898573

[34] O’Sullivan S. The age of diagnosis. Sickness, Health and Why Medicine Has Gone Too Far. Hodder & Stoughton, London: Hodder Press, 2025.

