# Supplementary material for: Multimorbidity trends in Catalonia, 2010–21: a population-based cohort study
Source: Int J Epidemiol. 2026 Jan 2;55(1):dyaf218. doi: 10.1093/ije/dyaf218 (PMC12758007; doi:10.1093/ije/dyaf218)
Supplement: dyaf218_Supplementary_Data [file dyaf218_supplementary_data.docx]

**Supplementary Material for**

**Multimorbidity dynamics in Catalonia, 2010-2021: a population-based cohort study**

Iñaki Permanyer

Jordi Gumà

Sergi Trias-Llimós

Aïda Solé-Auró

September 2025

**Table of contents**

[Table S1. List of diseases 2](#_Toc209605963)

[Table S2. Cohort specific logistic regression models for mortality (2010-2021). 12](#_Toc209605964)

[Figure S1 Lexis diagram illustrating the contribution of the birth cohort 1990-1999 to the prevalence and incidence of multimorbidity during the follow-up period (2010-2021). 15](#_Toc209605965)

[Figure S2. Median age at basic and complex multimorbidity onset by sex between 2010 and 2021. 16](#_Toc209605966)

[Figure S3. Trends in multimorbidity prevalence by age groups/cohorts (women and men separately) between 2010 and 2021. Common diseases with Head et al. (2021). 17](#_Toc209605967)

[Figure S4. Multimorbidity incidence by cohort and sex, according to basic and complex multimorbidity. Common diseases with Head et al. (2021) 18](#_Toc209605968)

## Table S1. List of diseases

| **ICD-10 Chapter** | **Chronic diseases** |
| --- | --- |
| **I Certain infectious and parasitic diseases** | A52: Late syphilis |
|  | B02: Herpes zoster |
|  | B18: Chronic viral hepatitis* |
|  | B24: Unspecified HIV disease |
| **II Neoplasms** | C00: Malignant neoplasm of lip |
|  | C01: Malignant neoplasm of base of tongue |
|  | C02: Malignant neoplasm of other and unspecified parts of tongue |
|  | C03: Malignant neoplasm of gum |
|  | C04: Malignant neoplasm of floor of mouth |
|  | C05: Malignant neoplasm of palate |
|  | C06: Malignant neoplasm of other and unspecified parts of mouth |
|  | C07: Malignant neoplasm of parotid gland |
|  | C08: Malignant neoplasm of other and unspecified major salivary glands |
|  | C09: Malignant neoplasm of tonsil |
|  | C10: Malignant neoplasm of oropharynx* |
|  | C11: Malignant neoplasm of nasopharynx |
|  | C12: Malignant neoplasm of pyriform sinus |
|  | C13: Malignant neoplasm of hypopharynx |
|  | C14: Malignant neoplasm of other and ill-defined sites in the lip, oral cavity, and pharynx |
|  | C15: Malignant neoplasm of esophagus* |
|  | C16: Malignant neoplasm of stomach* |
|  | C17: Malignant neoplasm of small intestine* |
|  | C18: Malignant neoplasm of colon* |
|  | C19: Malignant neoplasm of rectosigmoid junction |
|  | C20: Malignant neoplasm of rectum* |
|  | C21: Malignant neoplasm of anus and anal canal |
|  | C22: Malignant neoplasm of liver and intrahepatic bile ducts* |
|  | C23: Malignant neoplasm of gallbladder* |
|  | C24: Malignant neoplasm of other and unspecified parts of biliary tract* |
|  | C25: Malignant neoplasm of pancreas* |
|  | C26: Malignant neoplasm of other and ill-defined digestive organs |
|  | C30: Malignant neoplasm of nasal cavity and middle ear |
|  | C31: Malignant neoplasm of accessory sinuses |
|  | C32: Malignant neoplasm of larynx |
|  | C33: Malignant neoplasm of trachea |
|  | C34: Malignant neoplasm of bronchus and lung* |
|  | C37: Malignant neoplasm of thymus |
|  | C38: Malignant neoplasm of heart, mediastinum, and pleura |
|  | C39: Malignant neoplasm of other and ill-defined sites in the respiratory system |
|  | C40: Malignant neoplasm of bone and articular cartilage of limbs* |
|  | C41: Malignant neoplasm of bone and articular cartilage of other and unspecified sites* |
|  | C43: Malignant melanoma of skin* |
|  | C44: Other malignant neoplasms of skin* |
|  | C45: Mesothelioma* |
|  | C46: Kaposi’s sarcoma |
|  | C47: Malignant neoplasm of peripheral nerves and autonomic nervous system |
|  | C48: Malignant neoplasm of retroperitoneum and peritoneum |
|  | C49: Malignant neoplasm of other connective and soft tissue |
| **III Diseases of the blood and blood-forming organs and certain disorders involving the immune mechanism** | C50: Malignant neoplasm of breast* |
|  | C51: Malignant neoplasm of vulva |
|  | C52: Malignant neoplasm of vagina |
|  | C53: Malignant neoplasm of cervix uteri* |
|  | C54: Malignant neoplasm of corpus uteri* |
|  | C55: Malignant neoplasm of uterus, part unspecified* |
|  | C56: Malignant neoplasm of ovary* |
|  | C57: Malignant neoplasm of other and unspecified female genital organs |
|  | C60: Malignant neoplasm of penis |
|  | C61: Malignant neoplasm of prostate* |
|  | C62: Malignant neoplasm of testis* |
|  | C63: Malignant neoplasm of other and unspecified male genital organs |
|  | C64: Malignant neoplasm of kidney, except renal pelvis* |
|  | C65: Malignant neoplasm of renal pelvis |
|  | C66: Malignant neoplasm of ureter |
|  | C67: Malignant neoplasm of bladder* |
|  | C68: Malignant neoplasm of other and unspecified urinary organs |
|  | C69: Malignant neoplasm of eye and adnexa |
|  | C70: Malignant neoplasm of meninges |
|  | C71: Malignant neoplasm of brain* |
|  | C72: Malignant neoplasm of spinal cord, cranial nerves, and other parts of the central nervous system |
|  | C73: Malignant neoplasm of thyroid gland* |
|  | C74: Malignant neoplasm of adrenal gland |
|  | C75: Malignant neoplasm of other endocrine glands and related structures |
|  | C76: Malignant neoplasm of other and ill-defined sites* |
|  | C77: Secondary and unspecified malignant neoplasm of lymph nodes* |
|  | C78: Secondary malignant neoplasm of respiratory and digestive organs* |
|  | C79: Secondary malignant neoplasm of other sites* |
|  | C7A: Malignant neuroendocrine tumors |
|  | C7B: Secondary neuroendocrine tumors |
|  | C80: Malignant neoplasm without specification of site* |
|  | C81: Hodgkin lymphoma* |
|  | C82: Follicular lymphoma* |
|  | C83: Non-follicular lymphoma* |
|  | C84: Mature T/NK-cell lymphomas* |
|  | C85: Other and unspecified types of non-Hodgkin lymphoma* |
|  | C88: Malignant immunoproliferative diseases |
|  | C90: Multiple myeloma and malignant plasma cell neoplasms* |
|  | C91: Lymphoid leukemia* |
|  | C92: Myeloid leukemia* |
|  | C93: Monocytic leukemia* |
|  | C94: Other leukemias of specified cell type* |
|  | C95: Leukemia of unspecified cell type* |
|  | C96: Other and unspecified malignant neoplasms of lymphoid, hematopoietic, and related tissue |
|  | C97: Malignant neoplasms of independent (primary) multiple sites* |
|  | D29: Benign neoplasm of male genital organs |
|  | D50: Iron deficiency anemia |
|  | D51: Vitamin B12 deficiency anemia* |
|  | D52: Folate deficiency anemia* |
|  | D53: Other nutritional anemias |
|  | D55: Anemia due to enzyme disorders |
|  | D56: Thalassemia* |
|  | D57: Sickle-cell disorders* |
|  | D58: Other hereditary hemolytic anemias* |
|  | D59: Acquired hemolytic anemia* |
|  | D60: Acquired pure red cell aplasia |
|  | D61: Other aplastic anemias and bone marrow failure syndromes* |
|  | D62: Acute posthemorrhagic anemia |
|  | D63: Anemia in chronic diseases classified elsewhere |
|  | D64: Other anemias* |
|  | D65: Disseminated intravascular coagulation |
|  | D66: Hereditary factor VIII deficiency |
|  | D68: Other coagulation defects* |
|  | D69: Purpura and other hemorrhagic conditions* |
| **IV Endocrine, nutritional and metabolic diseases** | E00: Congenital iodine-deficiency syndrome |
|  | E01: Iodine-deficiency related thyroid disorders and allied conditions |
|  | E02: Subclinical iodine-deficiency hypothyroidism |
|  | E03: Other hypothyroidism* |
|  | E04: Other nontoxic goiter |
|  | E05: Thyrotoxicosis* |
|  | E06: Thyroiditis |
|  | E07: Other disorders of thyroid* |
|  | E10: Type 1 diabetes mellitus* |
|  | E11: Type 2 diabetes mellitus* |
|  | E13: Other specified diabetes mellitus* |
|  | E14: Unspecified diabetes mellitus* |
|  | E22: Hyperfunction of pituitary gland |
|  | E41: Nutritional marasmus |
|  | E43: Unspecified severe protein-calorie malnutrition |
|  | E44: Protein-calorie malnutrition of moderate and mild degree |
|  | E45: Retarded development following protein-calorie malnutrition |
|  | E46: Unspecified protein-calorie malnutrition |
|  | E66: Overweight and obesity |
|  | E78: Disorders of lipoprotein metabolism and other lipidemias |
|  | E86: Volume depletion |
|  | E87: Other disorders of fluid, electrolyte, and acid-base balance |
|  | E89: Postprocedural endocrine and metabolic disorders |
| **V Mental and behavioural disorders** | F00: Dementia in Alzheimer disease |
|  | F01: Vascular dementia* |
|  | F02: Dementia in other diseases classified elsewhere* |
|  | F03: Unspecified dementia* |
|  | F05: Delirium due to known physiological condition |
|  | F06: Other mental disorders due to known physiological condition |
|  | F10: Alcohol-related disorders* |
|  | F11: Opioid-related disorders* |
|  | F12: Cannabis-related disorders* |
|  | F13: Sedative, hypnotic, or anxiolytic-related disorders* |
|  | F14: Cocaine-related disorders* |
|  | F15: Other stimulant-related disorders* |
|  | F16: Hallucinogen-related disorders* |
|  | F17: Nicotine dependence* |
|  | F18: Inhalant-related disorders* |
|  | F19: Other psychoactive substance-related disorders* |
|  | F20: Schizophrenia* |
|  | F21: Schizotypal disorder |
|  | F22: Persistent delusional disorders |
|  | F23: Acute and transient psychotic disorders |
|  | F25: Schizoaffective disorders |
|  | F28: Other nonorganic psychotic disorders |
|  | F29: Unspecified nonorganic psychosis |
|  | F30: Manic episode |
|  | F31: Bipolar disorder |
|  | F32: Major depressive disorder, single episode |
|  | F33: Major depressive disorder, recurrent |
|  | F34: Persistent mood [affective] disorders |
|  | F40: Phobic anxiety disorders |
|  | F41: Other anxiety disorders |
|  | F42: Obsessive-compulsive disorder* |
|  | F43: Reaction to severe stress, and adjustment disorders |
|  | F70: Mild intellectual disabilities* |
|  | F71: Moderate intellectual disabilities* |
|  | F72: Severe intellectual disabilities* |
|  | F73: Profound intellectual disabilities* |
|  | F79: Unspecified intellectual disabilities* |
|  | F84: Pervasive developmental disorders* |
|  | F92: Mixed disorders of conduct and emotions |
|  | F93: Emotional disorders with onset specific to childhood |
| **VI Diseases of the nervous system** | G04: Encephalitis, myelitis, and encephalomyelitis |
|  | G11: Hereditary ataxia |
|  | G12: Spinal muscular atrophy and related syndromes* |
|  | G13: Systemic atrophy primarily affecting central nervous system in other diseases classified elsewhere |
|  | G20: Parkinson disease* |
|  | G21: Secondary parkinsonism |
|  | G22: Parkinsonism in other diseases classified elsewhere |
|  | G23: Other degenerative diseases of basal ganglia |
|  | G25: Other extrapyramidal and movement disorders* |
|  | G30: Alzheimer disease* |
|  | G31: Other degenerative diseases of nervous system, not elsewhere classified |
|  | G32: Other degenerative disorders of nervous system in diseases classified elsewhere |
|  | G35: Multiple sclerosis* |
|  | G36: Other acute disseminated demyelination |
|  | G37: Other demyelinating diseases of central nervous system |
|  | G40: Epilepsy and recurrent seizures* |
|  | G41: Status epilepticus |
|  | G43: Migraine |
|  | G45: Transient cerebral ischemic attacks and related syndromes* |
|  | G46: Vascular syndromes of brain in cerebrovascular diseases |
|  | G50: Disorders of trigeminal nerve |
|  | G51: Facial nerve disorders |
|  | G52: Disorders of other cranial nerves |
|  | G53: Cranial nerve disorders in diseases classified elsewhere |
|  | G54: Nerve root and plexus disorders |
|  | G55: Nerve root and plexus compressions in diseases classified elsewhere |
|  | G56: Mononeuropathies of upper limb |
|  | G57: Mononeuropathies of lower limb |
|  | G58: Other mononeuropathies |
|  | G59: Mononeuropathy in diseases classified elsewhere |
|  | G60: Hereditary and idiopathic neuropathy |
|  | G61: Inflammatory polyneuropathy |
|  | G62: Other polyneuropathies* |
|  | G63: Polyneuropathy in diseases classified elsewhere |
|  | G64: Other disorders of peripheral nervous system |
|  | G70: Myasthenia gravis and other myoneural disorders* |
|  | G80: Cerebral palsy* |
|  | G81: Hemiplegia |
|  | G82: Paraplegia and tetraplegia |
|  | G83: Other paralytic syndromes |
| **VII Diseases of the eye and adnexa** | H06: Disorders of lacrimal system |
|  | H28: Cataract in diseases classified elsewhere |
|  | H34: Retinal vascular occlusions* |
|  | H35: Other retinal disorders* |
|  | H36: Retinal disorders in diseases classified elsewhere |
|  | H40: Glaucoma* |
|  | H54: Visual impairment including blindness* |
| **VIII Diseases of the ear and mastoid process** | H81: Disorders of vestibular function* |
|  | H82: Vertiginous syndromes in diseases classified elsewhere |
|  | H90: Conductive and sensorineural hearing loss* |
|  | H91: Other hearing los* |
| **IX Diseases of the circulatory system** | I00: Rheumatic fever without mention of heart involvement |
|  | I01: Rheumatic fever with heart involvement |
|  | I02: Rheumatic chorea |
|  | I05: Rheumatic mitral valve diseases* |
|  | I06: Rheumatic aortic valve diseases* |
|  | I07: Rheumatic tricuspid valve diseases |
|  | I08: Multiple valve diseases* |
|  | I09: Other rheumatic heart diseases |
|  | I10: Essential (primary) hypertension |
|  | I11: Hypertensive heart disease |
|  | I12: Hypertensive chronic kidney disease |
|  | I13: Hypertensive heart and chronic kidney disease |
|  | I14: Secondary hypertension |
|  | I15: Secondary hypertension |
|  | I20: Angina pectoris* |
|  | I21: Acute myocardial infarction* |
|  | I22: Subsequent myocardial infarction* |
|  | I23: Certain current complications following myocardial infarction |
|  | I24: Other acute ischemic heart diseases |
|  | I25: Chronic ischemic heart disease* |
|  | I26: Pulmonary embolism* |
|  | I27: Other pulmonary heart diseases* |
|  | I28: Other diseases of pulmonary vessels |
|  | I30: Acute pericarditis |
|  | I31: Other diseases of pericardium |
|  | I32: Pericarditis in diseases classified elsewhere |
|  | I33: Acute and subacute endocarditis |
|  | I34: Nonrheumatic mitral valve disorders* |
|  | I35: Nonrheumatic aortic valve disorders* |
|  | I36: Nonrheumatic tricuspid valve disorders |
|  | I37: Pulmonary valve disorders |
|  | I38: Endocarditis, valve unspecified |
|  | I39: Endocarditis and heart valve disorders in diseases classified elsewhere |
|  | I40: Acute myocarditis |
|  | I41: Myocarditis in diseases classified elsewhere |
|  | I42: Cardiomyopathy* |
|  | I43: Cardiomyopathy in diseases classified elsewhere |
|  | I44: Atrioventricular and left bundle-branch block* |
|  | I45: Other conduction disorders* |
|  | I46: Cardiac arrest |
|  | I47: Paroxysmal tachycardia* |
|  | I48: Atrial fibrillation* |
|  | I49 - Other cardiac arrhythmias* |
|  | I50 - Heart failure* |
|  | I51 - Complications and ill-defined heart disease |
|  | I52 - Other heart disorders in diseases classified elsewhere |
|  | I60 - Nontraumatic subarachnoid hemorrhage* |
|  | I61 - Nontraumatic intracerebral hemorrhage* |
|  | I62 - Other nontraumatic intracranial hemorrhage |
|  | I63 - Cerebral infarction* |
|  | I64 - Stroke, not specified as hemorrhage or infarction |
|  | I65 - Occlusion and stenosis of precerebral arteries, not resulting in cerebral infarction |
|  | I66 - Occlusion and stenosis of cerebral arteries, not resulting in cerebral infarction |
|  | I67 - Other cerebrovascular diseases |
|  | I68 - Cerebrovascular disorders in diseases classified elsewhere |
|  | I69 - Sequelae of cerebrovascular disease |
|  | I70 - Atherosclerosis |
|  | I71 - Aortic aneurysm and dissection* |
|  | I72 - Other aneurysm |
|  | I73 - Other peripheral vascular diseases* |
|  | I74 - Arterial embolism and thrombosis |
|  | I77 - Other disorders of arteries and arterioles |
|  | I78 - Diseases of capillaries |
|  | I79 - Disorders of arteries, arterioles and capillaries in diseases classified elsewhere |
|  | I80 - Phlebitis and thrombophlebitis* |
|  | I81 - Portal vein thrombosis |
|  | I82 - Other venous embolism and thrombosis |
|  | I83 - Varicose veins of lower extremities |
|  | I85 - Esophageal varices* |
|  | I86 - Varicose veins of other sites |
|  | I87 - Other disorders of veins |
|  | I88 - Nonspecific lymphadenitis |
|  | I98 - Other disorders of circulatory system in diseases classified elsewhere |
|  | I99 - Other and unspecified disorders of circulatory system |
| **X Diseases of the respiratory system** | J41 - Simple and mucopurulent chronic bronchitis |
|  | J42 - Unspecified chronic bronchitis |
|  | J43 – Emphysema* |
|  | J44 - Other chronic obstructive pulmonary disease* |
|  | J45 - Asthma |
|  | J46 - Status asthmaticus |
|  | J47 – Bronchiectasis* |
|  | J60 - Coalworker's pneumoconiosis |
|  | J61 - Pneumoconiosis due to asbestos and other mineral fibers* |
|  | J62 - Pneumoconiosis due to dust containing silica |
|  | J63 - Pneumoconiosis due to other inorganic dusts |
|  | J64 - Unspecified pneumoconiosis |
|  | J66 - Airway disease due to specific organic dust |
|  | J67 - Hypersensitivity pneumonitis due to organic dust |
|  | J68 - Respiratory conditions due to inhalation of chemicals, etc. |
|  | J70 - Respiratory conditions due to other external agents |
|  | J84 - Other interstitial pulmonary diseases* |
|  | J92 - Pleural plaque |
| **XI Diseases of the digestive system** | K22 - Other diseases of esophagus* |
|  | K25 - Gastric ulcer |
|  | K26 - Duodenal ulcer |
|  | K27 - Peptic ulcer, site unspecified |
|  | K28 - Gastrojejunal ulcer |
|  | K29 - Gastritis and duodenitis |
|  | K50 - Crohn's disease* |
|  | K51 - Ulcerative colitis* |
|  | K55 - Vascular disorders of intestine* |
|  | K57 - Diverticular disease of intestine* |
|  | K70 - Alcoholic liver disease* |
|  | K71 - Toxic liver disease |
|  | K72 - Hepatic failure, not elsewhere classified* |
|  | K73 - Chronic hepatitis, not elsewhere classified |
|  | K74 - Fibrosis and cirrhosis of liver* |
|  | K76 - Other diseases of liver* |
| **XII Diseases of the skin and subcutaneous tissue** | L40 – Psoriasis* |
|  | L41 - Parapsoriasis |
|  | L89 - Pressure ulcer |
|  | L94 - Other localized connective tissue disorders |
| **XIII Diseases of the musculoskeletal system and connective tissue** | M05 - Rheumatoid arthritis with rheumatoid factor* |
|  | M06 - Other rheumatoid arthritis |
|  | M07 - Psoriatic and enteropathic arthropathies* |
|  | M08 - Juvenile arthritis |
|  | M09 - Juvenile arthritis in diseases classified elsewhere |
|  | M10 - Gout* |
|  | M11 - Other crystal arthropathies |
|  | M12 - Other specific arthropathies |
|  | M13 - Other arthritis |
|  | M14 - Arthropathies in other diseases classified elsewhere |
|  | M15 – Polyarthrosis* |
|  | M16 - Osteoarthritis of hip* |
|  | M17 - Osteoarthritis of knee* |
|  | M18 - Osteoarthritis of first carpometacarpal joint |
|  | M19 - Other and unspecified osteoarthritis* |
|  | M30 - Polyarteritis nodosa and related conditions |
|  | M31 - Other necrotizing vasculopathies* |
|  | M32 - Systemic lupus erythematosus* |
|  | M33 - Dermatopolymyositis |
|  | M34 - Systemic sclerosis* |
|  | M35 - Other systemic involvement of connective tissue* |
|  | M36 - Systemic disorders of connective tissue in diseases classified elsewhere |
|  | M45 - Ankylosing spondylitis* |
|  | M46 - Other inflammatory spondylopathies |
|  | M47 – Spondylosis* |
|  | M48 - Other spondylopathies* |
|  | M49 - Spondylopathies in diseases classified elsewhere |
|  | M50 - Cervical disc disorders |
|  | M51 - Other intervertebral disc disorders* |
|  | M53 - Other and unspecified dorsopathies, not elsewhere classified |
|  | M54 - Dorsalgia |
|  | M60 - Myositis |
|  | M61 - Calcification and ossification of muscle |
|  | M62 - Other disorders of muscle |
|  | M63 - Disorders of muscle in diseases classified elsewhere |
|  | M65 - Synovitis and tenosynovitis |
|  | M66 - Spontaneous rupture of synovium and tendon |
|  | M67 - Other disorders of synovium and tendon |
|  | M68 - Disorders of synovium and tendon in diseases classified elsewhere |
|  | M70 - Soft tissue disorders related to use, overuse, and pressure |
|  | M71 - Other bursopathies |
|  | M72 - Fibroblastic disorders* |
|  | M73 - Soft tissue disorders in diseases classified elsewhere |
|  | M75 - Shoulder lesions |
|  | M76 - Enthesopathies of lower limb, excluding foot |
|  | M77 - Other enthesopathies |
|  | M79 - Other soft tissue disorders, not elsewhere classified |
|  | M80 - Osteoporosis with current pathological fracture |
|  | M81 - Osteoporosis without current pathological fracture* |
|  | M82 - Osteoporosis in diseases classified elsewhere |
| **XIV Diseases of the genitourinary system** | N03 - Chronic nephritic síndrome* |
|  | N11 - Chronic tubulo-interstitial nephritis |
|  | N18 - Chronic kidney disease* |
|  | N19 - Unspecified kidney failure |
|  | N25 - Disorders resulting from impaired renal tubular function |
|  | N26 - Unspecified contracted kidney |
|  | N35 - Urethral stricture |
|  | N39 - Other disorders of urinary system* |
|  | N40 - Benign prostatic hyperplasia* |
|  | N41 - Inflammatory diseases of prostate |
|  | N42 - Other disorders of prostate |
|  | N51 - Disorders of male genital organs in diseases classified elsewhere |
|  | N95 - Menopausal and other perimenopausal disorders |

* Diseases also considered in the paper: Head A, Fleming K, Kypridemos C, Schofield P, Pearson-Stuttard J, O’Flaherty M. Inequalities in incident and prevalent multimorbidity in England, 2004–19: a population-based, descriptive study. *Lancet Healthy Longevity* 2021;2(8):e489–97.

## Table S2. Cohort specific logistic regression models for mortality (2010-2021).

*Cohort 1930-39*

|  |  | **Coeff.** | **Robust SE** | **Pr(>\|Z\|)** |
| --- | --- | --- | --- | --- |
| **Intercept** |  | -84.31 | 19.52 | *** |
| **Multimorbidity (Ref: No multimorbidity)** | Basic | -0.31 | 0.04 | *** |
|  | Complex | 0.28 | 0.04 | *** |
| **Year** |  | 0.04 | 0.01 | *** |
| **Gender (Ref: Men)** | Women | -0.44 | 0.05 | *** |
| **Sanitary region (Ref: Barcelona Ciutat)** | Metropolità Nord | 0.09 | 0.08 |  |
|  | Metropolità Sud | 0.05 | 0.09 |  |
|  | Catalunya Central | 0.16 | 0.09 | † |
|  | Lleida | 0.11 | 0.09 |  |
|  | Girona | 0.12 | 0.08 |  |
|  | Camp de Tarragona | 0.15 | 0.08 | † |
|  | Terres de l'Ebre | 0.08 | 0.09 |  |
|  | Alt Pirineu i Aran | 0.12 | 0.09 |  |

< 0.001 ***; <0.01**; <0.05*; <0.1†

*Cohort 1940-49*

|  |  | **Coeff.** | **Robust SE** | **Pr(>\|Z\|)** |
| --- | --- | --- | --- | --- |
| **Intercept** |  | -39.32 | 21.44 | † |
| **Multimorbidity (Ref: No multimorbidity)** | Basic | -0.50 | 0.04 | *** |
|  | Complex | 0.17 | 0.04 | *** |
| **Year** |  | 0.02 | 0.01 | † |
| **Gender (Ref: Men)** | Women | -0.78 | 0.06 | *** |
| **Sanitary region (Ref: Barcelona Ciutat)** | Metropolità Nord | 0.07 | 0.09 |  |
|  | Metropolità Sud | 0.03 | 0.10 |  |
|  | Catalunya Central | 0.06 | 0.10 |  |
|  | Lleida | 0.07 | 0.10 |  |
|  | Girona | 0.08 | 0.09 |  |
|  | Camp de Tarragona | 0.10 | 0.09 |  |
|  | Terres de l'Ebre | 0.10 | 0.10 |  |
|  | Alt Pirineu i Aran | 0.12 | 0.12 |  |

< 0.001 ***; <0.01**; <0.05*; <0.1†

*Cohort 1950-59*

|  |  | **Coeff.** | **Robust SE** | **Pr(>\|Z\|)** |
| --- | --- | --- | --- | --- |
| **Intercept** |  | 16.10 | 19.70 |  |
| **Multimorbidity (Ref: No multimorbidity)** | Basic | -0.22 | 0.05 | *** |
|  | Complex | 0.47 | 0.05 | *** |
| **Year** |  | -0.01 | 0.01 |  |
| **Gender (Ref: Men)** | Women | -0.82 | 0.05 | *** |
| **Sanitary region (Ref: Barcelona Ciutat)** | Metropolità Nord | -0.04 | 0.09 |  |
|  | Metropolità Sud | -0.02 | 0.09 |  |
|  | Catalunya Central | -0.01 | 0.09 |  |
|  | Lleida | -0.01 | 0.09 |  |
|  | Girona | -0.03 | 0.09 |  |
|  | Camp de Tarragona | 0.06 | 0.08 |  |
|  | Terres de l'Ebre | -0.08 | 0.10 |  |
|  | Alt Pirineu i Aran | 0.11 | 0.12 |  |

< 0.001 ***; <0.01**; <0.05*; <0.1†

*Cohort 1960-69*

|  |  | **Coeff.** | **Robust SE** | **Pr(>\|Z\|)** |
| --- | --- | --- | --- | --- |
| **Intercept** |  | 51.16 | 18.85 | *** |
| **Multimorbidity (Ref: No multimorbidity)** | Basic | 0.16 | 0.05 | *** |
|  | Complex | 0.93 | 0.05 | *** |
| **Year** |  | -0.03 | 0.01 | *** |
| **Gender (Ref: Men)** | Women | -0.71 | 0.05 | *** |
| **Sanitary region (Ref: Barcelona Ciutat)** | Metropolità Nord | 0.03 | 0.09 |  |
|  | Metropolità Sud | -0.04 | 0.09 |  |
|  | Catalunya Central | 0.06 | 0.10 |  |
|  | Lleida | 0.03 | 0.11 |  |
|  | Girona | 0.04 | 0.09 |  |
|  | Camp de Tarragona | 0.16 | 0.09 | † |
|  | Terres de l'Ebre | 0.11 | 0.12 |  |
|  | Alt Pirineu i Aran | 0.08 | 0.14 |  |

< 0.001 ***; <0.01**; <0.05*; <0.1†

*Cohort 1970-79*

|  |  | **Coeff.** | **Robust SE** | **Pr(>\|Z\|)** |
| --- | --- | --- | --- | --- |
| **Intercept** |  | 76.07 | 18.88 | *** |
| **Multimorbidity (Ref: No multimorbidity)** | Basic | 0.57 | 0.07 | *** |
|  | Complex | 1.24 | 0.07 | *** |
| **Year** |  | -0.04 | 0.01 | *** |
| **Gender (Ref: Men)** | Women | -0.55 | 0.06 | *** |
| **Sanitary region (Ref: Barcelona Ciutat)** | Metropolità Nord | 0.06 | 0.09 |  |
|  | Metropolità Sud | -0.04 | 0.09 |  |
|  | Catalunya Central | 0.14 | 0.11 |  |
|  | Lleida | 0.16 | 0.12 |  |
|  | Girona | 0.08 | 0.10 |  |
|  | Camp de Tarragona | 0.32 | 0.10 | *** |
|  | Terres de l'Ebre | 0.41 | 0.12 | *** |
|  | Alt Pirineu i Aran | 0.08 | 0.24 |  |

< 0.001 ***; <0.01**; <0.05*; <0.1†

*Cohort 1980-89*

|  |  | **Coeff.** | **Robust SE** | **Pr(>\|Z\|)** |
| --- | --- | --- | --- | --- |
| **Intercept** |  | 122.81 | 24.69 | *** |
| **Multimorbidity (Ref: No multimorbidity)** | Basic | 0.83 | 0.09 | *** |
|  | Complex | 1.33 | 0.11 | *** |
| **Year** |  | -0.07 | 0.01 | *** |
| **Gender (Ref: Men)** | Women | -0.72 | 0.08 | *** |
| **Sanitary region (Ref: Barcelona Ciutat)** | Metropolità Nord | -0.10 | 0.13 |  |
|  | Metropolità Sud | 0.12 | 0.12 |  |
|  | Catalunya Central | -0.34 | 0.20 | † |
|  | Lleida | 0.22 | 0.18 |  |
|  | Girona | 0.03 | 0.13 |  |
|  | Camp de Tarragona | 0.17 | 0.15 |  |
|  | Terres de l'Ebre | 0.37 | 0.22 | † |
|  | Alt Pirineu i Aran | -0.28 | 0.43 |  |

< 0.001 ***; <0.01**; <0.05*; <0.1†

*Cohort 1990-99*

|  |  | **Coeff.** | **Robust SE** | **Pr(>\|Z\|)** |
| --- | --- | --- | --- | --- |
| **Intercept** |  | 107.88 | 33.04 | *** |
| **Multimorbidity (Ref: No multimorbidity)** | Basic | 0.77 | 0.14 | *** |
|  | Complex | 1.42 | 0.15 | *** |
| **Year** |  | -0.06 | 0.02 | *** |
| **Gender (Ref: Men)** | Women | -0.74 | 0.11 | *** |
| **Sanitary region (Ref: Barcelona Ciutat)** | Metropolità Nord | -0.01 | 0.18 |  |
|  | Metropolità Sud | 0.06 | 0.19 |  |
|  | Catalunya Central | 0.30 | 0.24 |  |
|  | Lleida | 0.50 | 0.25 | * |
|  | Girona | 0.22 | 0.21 |  |
|  | Camp de Tarragona | 0.33 | 0.22 |  |
|  | Terres de l'Ebre | 0.16 | 0.40 |  |
|  | Alt Pirineu i Aran | 0.59 | 0.44 |  |

Figure S1. Lexis diagram illustrating the ages for which the birth cohort 1990-1999 contributes to the prevalence and incidence of multimorbidity during the follow-up period (2010-2021). Source: Authors’ elaboration.

In this Lexis diagram we illustrate what age groups are involved in the 1990-1999 birth cohort (i.e., the youngest one included in our analyses) during the period under study (2010-2021) as plotted in Figure 1. The area highlighted in grey indicates the time-age combinations that contribute to the multimorbidity prevalence curves depicted in that figure. As shown in the vertical axis of the Lexis diagram, the cohort born between 1990 and 1999 contributes to the multimorbidity prevalence curve between ages 11 and 31. Yet, the contributions are not the same across all ages. For instance, for age 11, we only have the individuals born in 1999 (who attained the age of 11 in 2010). For age 13, we have all the individuals born between 1997 and 1999 (who attained the age of 13 between years 2010 and 2012). For ages between 20 and 22, all the individuals of the 1990-1999 cohort contributed to the multimorbidity prevalence curve at some point between 2010 and 2021. However, for ages above 22, we again have less individuals contributing to that curve. For instance, for age 28, we only have the individuals born between 1990 and 1993 (who turned 28 between 2018 and 2021). Lastly, for age 31, we only have the individuals born in 1990 (who turned that age in 2021) that contributed to the curve.


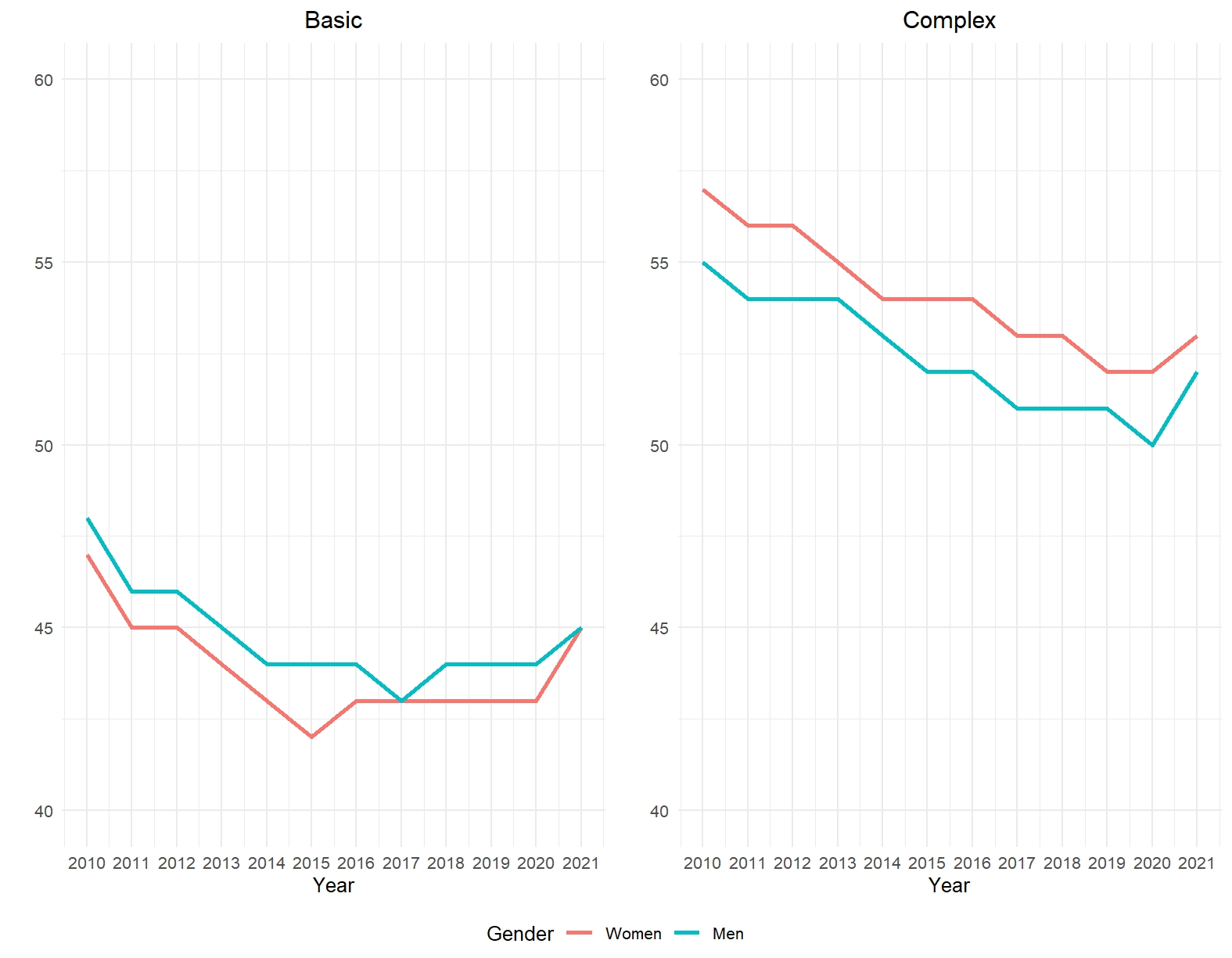


Figure S2. Median age at basic and complex multimorbidity onset by sex between 2010 and 2021. Source: Authors’ elaboration based on the HEALIN cohort database.


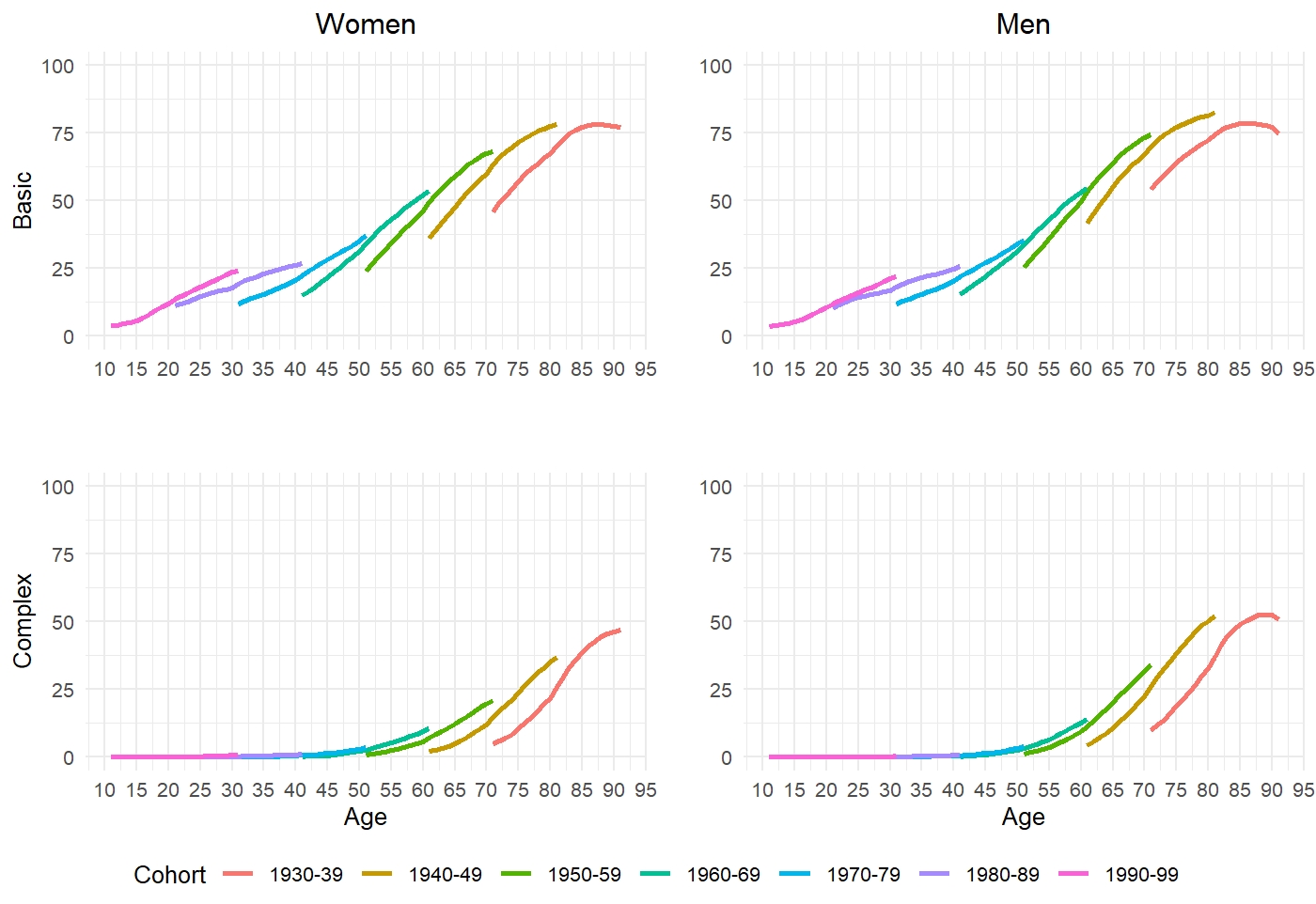


Figure S3. Trends in multimorbidity prevalence by age groups/cohorts (women and men separately) between 2010 and 2021. Common diseases with Head et al. (2021). *Source*: Authors’ elaboration based on the HEALIN cohort database.


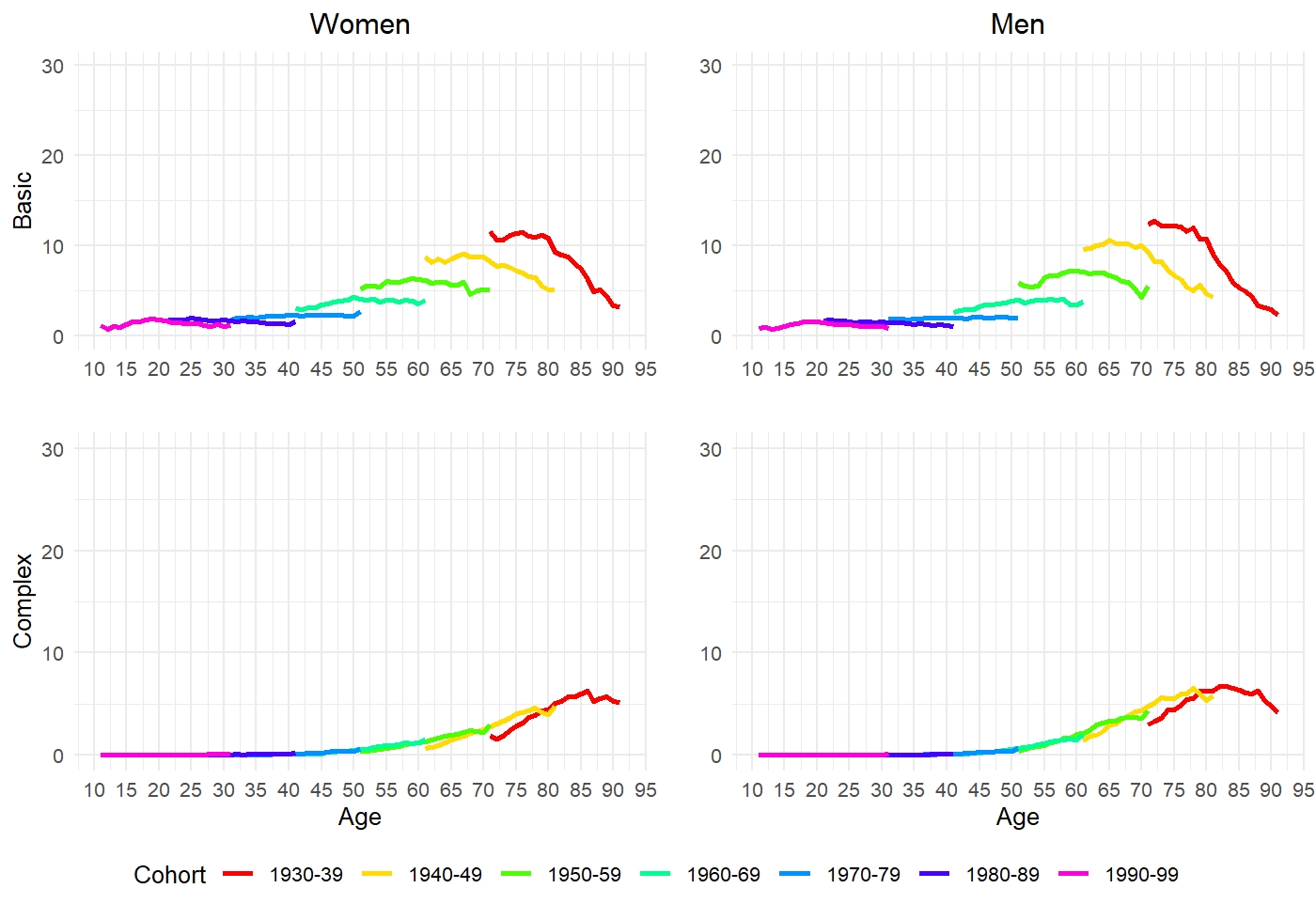


Figure S4. Multimorbidity incidence by cohort and sex, according to basic and complex multimorbidity. Common diseases with Head et al. (2021). *Source*: Authors’ elaboration based on the HEALIN cohort database.
